# Traditional Chinese herbal medicine for treating novel coronavirus (COVID-19) pneumonia: protocol for a systematic review and meta-analysis

**DOI:** 10.1186/s13643-020-01343-4

**Published:** 2020-04-08

**Authors:** Yuxi Li, Xiaobo Liu, Liuxue Guo, Juan Li, Dongling Zhong, Yonggang Zhang, Mike Clarke, Rongjiang Jin

**Affiliations:** 1 School of Acupuncture-Moxibustion and Tuina, Chengdu University of Traditional Chinese Medicine, Chengdu, China; 2 School of Health Preservation and Rehabilitation, Chengdu University of Traditional Chinese Medicine, Chengdu, China; 3 Hospital of Chengdu University of Traditional Chinese Medicine, Chengdu, China; 4 West China Hospital, Sichuan University, Chengdu, China; 5 Northern Ireland Clinical Trials Unit and Methodology Hub, Centre for Public Health, Queen’s University Belfast, Belfast, UK

**Keywords:** COVID-19, Coronavirus, Pneumonia, Traditional Chinese herbal medicine emerging infectious diseases, Systematic review, Meta-analysis

## Abstract

**Background:**

A new type of coronavirus, novel coronavirus (COVID-19), is causing an increasing number of cases of pneumonia and was declared a Public Health Emergency of International Concern by the World Health Organization on 30 January 2020. The virus first appeared in Wuhan, China, in late December 2019, and traditional Chinese herbal medicine is being used for its treatment. This systematic review and meta-analysis will assess studies of the effects of traditional Chinese herbal medicine in COVID-19 pneumonia.

**Methods:**

We will search electronic databases including PubMed, Embase, the Cochrane Central Register of Controlled Trials (CENTRAL), Chinese Biomedical Literature Database (CBM), China National Knowledge Infrastructure (CNKI), Chinese Science and Technology Periodical Database (VIP), and Wanfang database using keywords related to COVID-19 and traditional Chinese herbal medicine. Reference lists of relevant trials and reviews will be searched. We will manually search gray literature, such as conference proceedings and academic degree dissertations, and trial registries. Two independent reviewers will screen studies (XL and DZ), extract data (YL and LG), and evaluate risk of bias (YL and DZ). Data analysis will be conducted using the Review Manager software (version 5.3.5) and R software (version 3.6.1). Statistical heterogeneity will be assessed using a standard chi-square test with a significance level of *P* < 0.10. Biases associated with study size (e.g., publication bias) will be investigated using funnel plots, Egger’s test and Begg’s test, and Trim and Fill analysis.

**Discussion:**

This study will provide a high-quality synthesis of the effects of traditional Chinese herbal medicine for COVID-19. The use of traditional Chinese herbal medicine for treatment or prevention of these novel viral infections affecting the pneumonia will be investigated.

**Systematic review registration:**

PROSPERO registration number: CRD42020168004

## Background

Recently, a new type of coronavirus was identified and named 2019 novel coronavirus (COVID-19) by the World Health Organization (WHO) [1]. It has been causing an increasing rate of pneumonia cases since late December 2019 [2–4]. Infections were first identified in Wuhan, China, before being detected in other Chinese cities and in more than a dozen countries around the world by early February 2020 [5]. The outbreak was declared a Public Health Emergency of International Concern by the WHO on 30 January 2020. COVID-19-infected pneumonia is characterized by flu-like symptoms including fever, cough, severe acute respiratory distress syndrome, and in some cases death [6–8]. Human-to-human transmission has been confirmed for the virus [9–11], which is considered related to severe acute respiratory syndrome (SARS) and the Middle East respiratory syndrome (MERS). Like SARS-CoV and MERS-CoV, the COVID-19 is a serious threat to human health [7, 12]. As of 16 March 2020, nearly 170,000 people have been diagnosed with COVID-19 in the world [13]. Effective prevention and treatment are crucial in this situation.

Traditional Chinese herbal medicine therapy is a mixture of Chinese herbs prescribed by Chinese herbalists depending on the differentiation of the patient’s syndrome according to Chinese diagnostic patterns (inspection, listening, smelling, inquiry, and palpation). Studies have reported that Chinese herbal formula, such as San Wu Huangqin Decoction, Lianhuaqingwen Capsule, and Yinhuapinggan granule, possesses antiviral effects, which might be associated with blocking of the proliferation and replication of the viral particles, and that they might be able to improve lung damage by influenza viruses [14–16]. During the SARS epidemics, traditional Chinese herbal medicine treatments were reported to have successfully prevented and treated SARS [17–19]. Furthermore, traditional Chinese herbal medicine combined with western medicine treatment regimen reduced adverse events and other complications induced by glucocorticoid, antibiotic, and antiviral treatments [20, 21].

After the pneumonia outbreak with COVID-19, the State Administration of Traditional Chinese Medicine in China led an expert team to formulate a traditional Chinese herbal medicine treatment program. On 24 January 2020, the first case of a cured patient in Beijing being discharged from hospital after traditional Chinese herbal medicine treatment with symptomatic therapy was reported [22]. Later, another cured patient was reported following traditional Chinese herbal medicine therapy, prompting the wider application of traditional Chinese herbal medicine for patients with COVID-19 pneumonia [23]. On 27 January 2020, the General Office of the National Health and Health Commission of China and the Office of the State Administration of Traditional Chinese Medicine issued “Diagnosis and Treatment of Pneumonia Caused by Novel Coronavirus Infection (Trial Version 4).” This included the updated traditional Chinese herbal medicine treatment program and required local health and health committees to implement and strengthen the integration of traditional Chinese herbal medicine and western medicine [24].

Although traditional Chinese herbal medicine treatment is being applied for COVID-19 pneumonia, uncertainty remains about its effectiveness. Therefore, we intend to systematically review studies of the application of traditional Chinese herbal medicine in COVID-19 patients in order to examine the empirical evidence of the effects of traditional Chinese herbal medicine for COVID-19 pneumonia. We aim to provide a robust evidence base for clinical practice in treating COVID-19 pneumonia.

## Methods/design

### Study registration

This systematic review was registered on PROSPERO (CRD42020168004) on 5 February 2020. We have prepared this protocol in accordance with the Preferred Reporting Item for Systematic Review and Meta-analysis (PRISMA-P) statement [25] (Additional file 1), and we will update the PROSPERO record if there are any important amendments.

### Eligibility criteria

#### Inclusion criteria

##### Type of studies

Randomized trials and quasi-randomized or prospective controlled clinical trials that have tested traditional Chinese herbal medicine with or without western medicine for COVID-19 will be included. There will be no restrictions for blinding, follow-up, or publication status. Publications in English and Chinese will be included.

##### Type of participant

Patients diagnosed with pneumonia caused by COVID-19 without immediately life-threatening co-morbidities will be included. There will be no restrictions with respect to gender, age, or ethnicity.

##### Type of interventions

Traditional Chinese herbal medicine involving extracts from herbs, single or mixture herbal formulas regardless of their compositions or forms. Traditional Chinese herbal medicine combined with one or more other pharmacological intervention will also be included. There will be no restrictions with respect to dosage, frequency, duration, or follow-up time of treatment.

##### Type of comparators

There will be no restrictions with respect to the type of comparator. The comparators are likely to include western medical therapies, supportive care, and other therapeutic methods.

##### Type of outcome measurements

Our primary outcomes will be survival at the end of treatment and at the end of follow-up, and time and rate of the patient becoming negative for the COVID-19. We will also assess the following outcomes at the end of treatment and at the end of follow-up: days to absence of fever, symptom score (based on fever, fatigue, cough, difficulty in breathing, poor appetite, etc.), duration of each symptom, pulmonary function, inflammation index, results of chest computerized tomography, length of stay in hospital, use (including dosage and duration) of corticosteroid, quality of life, and adverse events. If other outcomes are reported in the eligible studies, these will be extracted and reported but we will give particular attention to the possibility of selective reporting bias when using any such outcomes in our review.

#### Exclusion criteria

The exclusion criteria are as follows: (1) patients with life-threatening co-morbidities likely to lead to death within the trial follow-up period, (2) duplicated data or data that cannot be extracted after contacting original authors, and (3) full text cannot be obtained after contacting original authors.

### Databases and search strategy

We will search electronic databases including PubMed, Embase, the Cochrane Central Register of Controlled Trials (CENTRAL), Chinese Biomedical Literature Database (CBM), China National Knowledge Infrastructure (CNKI), Chinese Science and Technology Periodical Database (VIP), and Wanfang database (Wanfang Data) using keywords combination, such as novel coronavirus OR COVID-19 OR 2019-nCoV OR COVID-2019 pneumonia AND traditional Chinese herbal medicine OR Chinese herb OR traditional Chinese medicine. The full search strategy for PubMed is provided in Additional file 2, and similar strategies will be applied to the other electronic databases. Reference lists of relevant trials and reviews will be searched. We will manually search gray literature such as conference proceedings and academic degree dissertations, and trial registries (both through the WHO International Clinical Trials Registry Platform (ICRP) and on the websites of national registries). We will consult experts in the field for possible studies to be included.

### Study selection

We will export the identified records in databases into EndNote X9 software and use this to identify duplicates. After removing duplicates, the retrieved records will be checked independently by two reviewers (XL and DZ), who will apply the eligibility criteria based on the title and abstract. Where a study is potentially eligible, the full text will be obtained and checked independently by two reviewers (XL and DZ) to identify the eligible studies. Any disagreements will be discussed and resolved in discussion with a third reviewer (JL).

### Data extraction

In order to achieve a consistency (at least 80%) of extracted items, the data extractors will extract data from a sample of eligible studies. Results of the pilot extraction will be discussed among review authors and extractors. Two independent reviewers (YL and LG) will extract data with a predefined extraction template, which includes the following items: (1) general information—first author, title, journal, year of publication, country, funding source, study design, etc.; (2) characteristics of patients—age, gender, stage and severity of disease, syndrome differentiation, comorbidity, etc.; (3) characteristics of intervention—protocol of Chinese herbal medicine (types, dosage, frequency, duration, etc.) and protocol of comparators (types, dosage, frequency, duration, etc.); (4) characteristics of trial—study setting (ambulatory sector/hospital), sample size (numbers recruited, randomized, or allocated to the interventions by another method, followed up and analyzed), generation of randomization sequence, allocation concealment, blinding, studies’ length of follow-up, etc.; and (5) outcomes—all outcomes, main conclusions, adverse events, etc. The original authors will be contacted to request missing data where necessary. Extracted information will be cross-checked by YL and LG. Any disagreements will be discussed and resolved in discussion with a third reviewer (YZ).

### Assessment of risk of bias

In order to achieve a consistency (at least 80%) of risk of bias assessment, the risk of bias assessors will pre-assess a sample of eligible studies. Results of the pilot risk of bias will be discussed among review authors and assessors. Two independent reviewers (YL and DZ) will assess the risk of bias of the included studies at study level. We will follow the guidance in the latest version of *Cochrane Handbook for systematic reviews of interventions* [26] when choosing and using tools to assessing risk of bias for randomized trials (version 2 of the Cochrane risk-of-bias tool for randomized trials, RoB 2 [27]) and non-randomized trials (the Risk Of Bias In Non-randomized Studies of Interventions, ROBINS-I tool [28]). Any disagreements will be discussed and resolved in discussion with a third reviewer (RJ). Studies with high risk of bias or unclear bias will be given less weight in our data synthesis.

### Data analysis

Statistical analyses will be conducted using the RevMan software (version 5.3.5) and R software (version 3.6.1). If possible, analyses for all outcomes will be done by intention to treat. We will perform analyses to provide effect estimates for dichotomous data and continuous data, with 95% confidence intervals. We will use risk ratios (RR) for dichotomous data and mean differences (MD) for continuous data. We will explore the heterogeneity before we perform meta-analysis for outcomes. Heterogeneity will be detected by using a standard chi-square test with a significance level of *P* < 0.10. The *I*^2^ statistic will be applied to quantify inconsistency across studies and to assess the impact of heterogeneity on the meta-analyses. The Mantel-Haenszel method will be used for dichotomous outcomes, and the DerSimonian and Laird inverse variance method will be used for continuous outcomes. Random-effects model will be used to pool the data.

### Subgroup analysis

If an adequate number of studies are identified, we will perform subgroup analysis for the following variables: age and patients with or without other diseases and COVID-19 stage at which the traditional Chinese herbal medicine was given.

We will also consider analyses for other subgroups as reported in the included studies, but we will give particular attention to the possibility of selective reporting bias when using any such subgroups in our review.

### Trial sequential analysis

Trial sequential analysis provides the necessary sample size for our meta-analysis and boundaries that determine whether the evidence in our meta-analysis is reliable and conclusive [29, 30]. We will perform a trial sequential analysis to maintain an overall 5% risk of type 1 error and calculate the required sample size.

### Sensitivity analysis

To check the robustness of pooled outcome results, we will carry out sensitivity analysis to explore the influence of studies with high risk of bias.

### Publication bias

If a sufficient number of articles are included, we will assess small study biases (e.g., publication bias) with funnel plots, Egger’s test and Begg’s test, and Trim and Fill analysis.

### Quality of evidence

Two independent reviewers (DLZ and JL) will assess the quality of evidence for each outcome with the Grading of Recommendations Assessment, Development, and Evaluation (GRADE) system [31]. Each outcome will be assessed for each of the five aspects: limitations, inconsistency, indirectness, imprecision, and publication bias. They will be rated as high, moderate, low, or very low level.

## Discussion

Like the outbreaks caused by SARS and MERS, the recent outbreak of COVID-19 in China is creating a substantial public health challenge. In 2002, traditional Chinese herbal medicine played an important role in the treatment of SARS, and 58.3% of confirmed cases received traditional Chinese herbal medicine [32]. A Cochrane Review [33] found that Chinese herbs combined with western medicine significantly improved symptoms of SARS, including decreasing body temperature, cough and breathing difficulties, dosages of corticosteroids, improving absorption of pulmonary infiltration, and quality of life. A review [34] of 90 studies of traditional Chinese herbal medicine for SARS revealed positive but inconclusive results about the efficacy of combined treatment, using traditional Chinese herbal medicine as an adjuvant. Based on previous experience of treating SARS with traditional Chinese herbal medicine, the General Office of the National Health and Health Commission of China and the Office of the State Administration of Traditional Chinese Medicine encouraged the integration of traditional Chinese herbal medicine and western medicine. Different prescriptions are recommended in different stages of disease.

This is the first systematic review to examine empirical evidence of the application of traditional Chinese herbal medicine for COVID-19 pneumonia. It will provide an overview of the application of traditional Chinese herbal medicine for treating COVID-19 patients and assess the strengths and limitations of available evidence. Furthermore, it will be guided by the PRISMA statement [25] and A MeaSurement Tool to Assess systematic Reviews (AMSTAR) 2 checklist [35] to achieve as high a level of quality as possible in reporting and methodology. This protocol may have the limitation that we developed it based on designs of current registered clinical trials, but we will update our proposed methods promptly on our PROSPERO record should these methods change. This review will help explore the potential role for traditional Chinese herbal medicine in the treatment or prevention of viral infections affecting the pneumonia.

## Supplementary information

**Additional file 1.** PRISMA-P 2015 Checklist.

**Additional file 2.** Search Strategy for PubMed.

## Data Availability

Not applicable.

## Abbreviations

CBM: Chinese Biomedical Literature Database
CENTRAL: Cochrane Central Register of Controlled Trials
CNKI: China National Knowledge Infrastructure
COVID-19: The novel coronavirus
MERS: Middle East respiratory syndrome
PROSPERO: Prospective Register of Systematic Reviews
SARS: Severe acute respiratory syndrome
VIP: Chinese Science and Technology Periodical Database
