# Supplementary material for: Traditional Chinese herbal medicine for treating novel coronavirus (COVID-19) pneumonia: protocol for a systematic review and meta-analysis
Source: Syst Rev. 2020 Apr 8;9:75. doi: 10.1186/s13643-020-01343-4 (PMC7138957; doi:10.1186/s13643-020-01343-4)
Supplement: Supplementary file 2 — Additional file 2. Search Strategy for PubMed. [file 13643_2020_1343_MOESM2_ESM.docx]

**Additional file 2 Search Strategy for PubMed**

| **#1** | Search novel coronavirus[Title/Abstract] |
| --- | --- |
| **#2** | Search COVID-2019[Title/Abstract] |
| **#3** | Search 2019-nCoV[Title/Abstract] |
| **#3** | Search novel coronavirus pneumonia[Title/Abstract] |
| **#4** | Search COVID-2019 pneumonia[Title/Abstract] |
| **#5** | Search 2019-nCoV pneumonia[Title/Abstract] |
| **#6** | Search #1 OR #2 OR #3 OR #4 OR #5 |
| **#7** | Search traditional Chinese medicine[MeSH Terms] |
| **#8** | Search Drugs, Chinese Herbal[MeSH Terms] |
| **#9** | Search traditional Chinese herbal medicine[Title/Abstract] |
| **#10** | Search Chinese herb*[Title/Abstract] |
| **#11** | Search #7 OR #8 OR #9 OR #10 |
| **#12** | Search #6 AND #11 |
